# Pathways to resilience: relationships between cognitive reserve, psychological debt, and Alzheimer’s disease biomarkers

**DOI:** 10.1186/s13195-026-02054-z

**Published:** 2026-04-24

**Authors:** Hanna Boscheck, Maxie Luft, René Mauer, Selin Senguel, Ylenia D’elia, Peter Dechent, Klaus Fliessbach, Wenzel Glanz, Stefan Hetzer, Daniel Janowitz, Ingo Kilimann, Luca Kleineidam, Marie Kronmüller, Falk Lüsebrink, Lukas Preis, Boris Rauchmann, Ayda Rostamzadeh, Björn Hendrik Schott, Sebastian Sodenkamp, Eike Spruth, Sophia Stoecklein, Renat Yakupov, Gabriel Ziegler, Frederic Brosseron, Katharina Buerger, Julian Hellmann-Regen, Christoph Laske, Robert Perneczky, Oliver Peters, Josef Priller, Alfredo Ramirez, Anja Schneider, Annika Spottke, Stefan Teipel, Jens Wiltfang, Frank Jessen, Emrah Düzel, Sandra Röske, Michael Wagner, Franka Glöckner, Natalie L. Marchant, Olga Klimecki, Miranka Wirth, DELCODE study group

**Affiliations:** 1 German Center for Neurodegenerative Diseases (DZNE), Standort Dresden, Tatzberg 41, Dresden, 01307 Germany; 2 Faculty of Medicine Carl Gustav Carus, Institute for Medical Informatics and Biometry (IMB), Technische Universität Dresden, Dresden, Germany; 3 Center for the Neuroscience of Embodied Cognition (CeNEC) at the Brain and Creativity Institute, University of Southern California, Los Angeles, CA USA; 4 Department of Cognitive Neurology, MR-Research in Neurosciences, University Medical Center Göttingen, Göttingen, Germany; 5 German Center for Neurodegenerative Diseases (DZNE), Bonn, Germany; 6 Clinic for Geriatric Psychiatry and Cognitive Disorders, University Hospital Bonn and University of Bonn, Bonn, Germany; 7 German Center for Neurodegenerative Diseases (DZNE), Magdeburg, Germany; 8 Berlin Center for Advanced Neuroimaging, Charité – Universitätsmedizin Berlin, Berlin, Germany; 9 Institute for Stroke and Dementia Research (ISD), University Hospital, LMU Munich, Munich, Germany; 10 German Center for Neurodegenerative Diseases (DZNE), Rostock, Germany; 11 Department of Psychosomatic Medicine, Rostock University Medical Center, Rostock, Germany; 12 Institute of Cognitive Neurology and Dementia Research (IKND), Otto-Von-Guericke University, Magdeburg, Germany; 13 Department of Psychiatry and Neurosciences, Charité Universitätsmedizin Berlin, Berlin, Germany; 14 Department of Psychiatry and Psychotherapy, University Hospital, LMU Munich, Munich, Germany; 15 Sheffield Institute for Translational Neuroscience (SITraN), University of Sheffield, Sheffield, UK; 16 Department of Neuroradiology, University Hospital LMU, Munich, Germany; 17 Department of Psychiatry, University of Cologne, Medical Faculty, Cologne, Germany; 18 German Center for Neurodegenerative Diseases (DZNE), Goettingen, Germany; 19 Department of Psychiatry and Psychotherapy, University Medical Center Goettingen, Goettingen, Germany; 20 Department of Psychiatry and Psychotherapy, Otto-Von-Guericke University, Magdeburg, Germany; 21 German Center for Neurodegenerative Diseases (DZNE), Tübingen, Germany; 22 Department of Psychiatry and Psychotherapy, University of Tübingen, Tübingen, Germany; 23 German Center for Neurodegenerative Diseases (DZNE), Berlin, Germany; 24 Department of Psychiatry and Psychotherapy, Charité, Berlin, Germany; 25 Department of Radiology, Ludwig Maximilian University Hospital, Munich, Germany; 26 German Center for Neurodegenerative Diseases (DZNE), Munich, Germany; 27 Charité Universitätsmedizin Berlin, ECRC Experimental and Clinical Research Center, Berlin, Germany; 28 Hertie Institute for Clinical Brain Research and Department of Psychiatry and Psychotherapy, Section for Dementia Research, University of Tübingen, Tübingen, Germany; 29 Munich Cluster for Systems Neurology (SyNergy) Munich, Munich, Germany; 30 Ageing Epidemiology Research Unit (AGE), School of Public Health, Imperial College London, London, UK; 31 University of Edinburgh and UK DRI, Edinburgh, UK; 32 Department of Psychiatry and Psychotherapy, School of Medicine and Health, Technical University of Munich, and German Center for Mental Health (DZPG), Munich, Germany; 33 Cologne Excellence Cluster On Cellular Stress Responses in Aging Associated Diseases (CECAD), University of Cologne, Cologne, Germany; 34 Department of Psychiatry and Psychotherapy, Division of Neurogenetics and Molecular Psychiatry, Faculty of Medicine and University Hospital Cologne, University of Cologne, Cologne, Germany; 35 Department of Psychiatry & Glenn Biggs Institute for Alzheimer’s and Neurodegenerative Diseases, San Antonio, TX USA; 36 Clinic for Parkinson’s, Sleep and Movement Disorders, Centre for Neurology, University Hospital Bonn, Bonn, Germany; 37 Department of Medical Sciences, Neurosciences and Signaling Group, Institute of Biomedicine (iBiMED), University of Aveiro, Aveiro, Portugal; 38 Faculty of Psychology, Chair of Lifespan Developmental Neuroscience, TU Dresden, Dresden, Germany; 39 Division of Psychiatry, University College London, London, UK; 40 Biological Psychology, University of Innsbruck, Innsbruck, Austria

**Keywords:** Alzheimer’s disease (AD), Biomarkers, Healthy aging, Prevention, Structural equation modeling, Sex differences, APOE ε4 genotype

## Abstract

**Background:**

Potentially modifiable lifestyle and psychological factors may influence Alzheimer’s disease (AD)-related brain pathology and cognitive function, thereby influencing cognitive resilience in late life.

**Objective:**

This cross-sectional study investigated associations and pathways between lifestyle and psychological factors related to cognitive reserve and psychological debt, AD-related biomarkers, and cognitive function, as well as potential differences in these associations between AD risk groups.

**Methods:**

In total, 298 non-demented older adults (mean age = 69.5 years, 44% women) of the DELCODE study were included. Structural equation modeling was used to assess the associations between the constructs of cognitive reserve (education, occupational complexity, leisure activity participation) and psychological debt (depression and anxiety symptoms, neuroticism, sleep quality), manifest AD-related biomarkers (cerebrospinal fluid [CSF] amyloid-beta [Aβ] 42, splenial white matter hyperintensities [WMH], hippocampal volume), and latent cognitive function of increased AD risk (Preclinical Alzheimer’s Cognitive Composite [PACC]). In the structural equation model, biomarkers were transformed such that higher values indicated greater AD-related brain pathology and age was included as a covariate. Multigroup analyses assessed moderations by established AD risk modifiers, namely sex and apolipoprotein ε4 (APOE ε4) genotype.

**Results:**

In the total sample, higher cognitive reserve was associated with better cognitive function (*p* = .005), independent of AD-related biomarkers. Higher cognitive reserve was associated with lower psychological debt (*p* = .035); however, neither construct showed a significant association with the AD-related biomarkers (*p* ≥ .177). AD-related biomarkers of CSF Aβ42 (*p* = .021), splenial WMH (*p* = .044), and hippocampal neurodegeneration (*p* = .007) were each independently associated with lower cognitive function. Most associations were comparable between AD risk groups stratified by sex and APOE ε4 genotype. The relationships between cognitive reserve and psychological debt, and between CSF Aβ42 and splenial WMH were stronger in APOE ε4 non-carriers than in carriers (all *p* ≤ .020).

**Conclusions:**

Cognitive reserve emerges as a key resilience pathway, supporting late-life cognition independently of AD-related pathology, with largely consistent effects across AD risk groups. The role of psychological debt warrants longitudinal investigation, particularly in vulnerable older populations.

**Trial registration:**

German Clinical Trials Register: DRKS00007966, Registered: 4 May 2015.

**Supplementary Information:**

The online version contains supplementary material available at 10.1186/s13195-026-02054-z.

## Introduction

The increase in Alzheimer’s disease (AD) and limited pharmacological treatment options highlight the need for effective prevention strategies. Targeting modifiable lifestyle and psychological risk factors offers a promising approach [1]. Few studies have investigated how these factors are associated with AD-related brain pathology and cognitive function in older adults.

Lifestyle protective and psychological risk factors can enhance resilience or vulnerability to cognitive decline and AD [1], as conceptualized by the constructs of cognitive reserve [2] and cognitive debt [3]; the latter was recently reframed as psychological debt (personal communication with N.L. Marchant, February 27, 2026). Cognitive reserve refers to the enhancement of cognitive resources through lifestyle protective factors, including higher education, occupational complexity, and long-term engagement in leisure activities, which are consistently linked to greater brain and cognitive health [4–7]. Conversely, psychological debt refers to adverse psychological risk factors, including clinical mental health symptoms, personality traits, and stressful life experiences, that increase the vulnerability to cognitive decline and AD development [8, 9]. The neurobiological mechansims and pathways linking cognitive reserve and psychological debt to cognitive health and resilience in late life remain unclear.

According to previous findings, lifestyle and psychological factors may directly influence AD-related brain pathology. Protective factors, such as cognitive engagement and positive psychological traits, are associated with lower amyloid-beta (Aβ) pathology and larger hippocampal volumes [5, 10–12]. In contrast, lower levels of reserve factors, such as lower educational attainment, as well as higher levels of risk factors, such as repetitive negative thinking and neuroticism, have been linked to Aβ pathology and tau burden [13–15], while findings for anxiety and depression are inconsistent [16, 17]. In addition, white matter hyperintensities (WMH) in the splenium of the corpus callosum have been proposed as a potential biomarker of AD-related brain pathology [18]. Splenial WMH have been identified as a convergence point of Aβ and vascular pathology and are associated with cognitive decline [19–21]. The relationships between multiple AD-related biomarkers and both lifestyle protective and psychological risk factors warrant further investigation.

Given the multifactorial nature of AD, assessing isolated lifestyle protective and psychological risk factors may not adequately capture the complexity of the disease. Structural equation modeling (SEM) offers a powerful tool to investigate the direct and indirect associations and pathways across multiple lifestyle protective and psychological risk factors, multiple AD-related biomarkers, and cognitive function in older adults in one integrated path model. In prior SEM studies, higher engagement in cognitive, physical, and social activities, as well as lower vascular/metabolic risk have been linked to better cognitive function [22–26], partially mediated by lower (hippocampal) neurodegeneration and lower white matter lesions [24–26].

Sex and apolipoprotein ε4 (APOE ε4) genotype have been identified as important epidemiological and genetic modifiers of AD risk and need to be considered in resilience and pathological pathways. In particular, women and APOE ε4 carriers are at greater risk for developing AD [1, 7, 27], potentially moderating the effects of lifestyle and psychological factors on brain and cognitive health in older adults. Sex differences in lifestyle, AD-related biomarkers, and cognitive function and their mutual relationships have been observed [23, 28–30]. In particular, women with Aβ pathology may show more pronounced hippocampal atrophy and faster cognitive decline [29]. APOE ε4 carriers typically show greater Aβ pathology [23, 25, 31] and appear to be more sensitive to influences of potentially modifiable lifestyle factors [24, 31, 32].

Building on previous work [6, 22, 23, 26], this cross-sectional SEM study investigated an integrated path model to assess how lifestyle-related protective factors (cognitive reserve) and psychological risk factors (psychological debt) are associated with late-life cognitive function. We further assessed whether these relationships were mediated by AD-related brain pathology (measured using cerebrospinal fluid [CSF] Aβ42, splenial WMH, and hippocampal volume), and whether these associations differed by known AD risk modifiers, as defined by sex and APOE ε4 genotype. Cognitive reserve, psychological debt, and cognitive function were modeled as constructs (latent variables) in non-demented older adults from the DELCODE study [33]. In the structural equation model, the AD-related biomarkers were transformed such that higher values consistently reflected greater AD-related brain pathology and age was included as a potential confounder. We hypothesized that 1.) cognitive reserve and psychological debt would be associated with cognitive function; 2.) AD-related biomarkers would mediate these associations; and 3.) these relationships would differ by established AD risk modifiers.

## Methods

The present study investigated the baseline dataset of the DELCODE study (DRKS00007966) [33], which is an observational longitudinal memory-clinic based multi-center study, investigating participants across different AD-risk stages (healthy controls, participants with subjective cognitive decline [SCD], mild cognitive impairment [MCI], family history of AD). The DELCODE study baseline dataset contains assessment of clinical and neuropsychological testing, magnetic resonance imaging (MRI), blood and urine sampling, as well as CSF assessments with annual follow-ups for a minimum of five years [33]. Detailed information is provided in the DELCODE study protocol [33].

### Participants

The DELCODE study baseline dataset comprises *N* = 1079 participants. In the present study, only participants with available AD-related biomarker data (CSF Aβ42, splenial WMH, and hippocampal neurodegeneration), as well as data in the variables of interest to model the constructs (also referred to as latent variables) cognitive reserve, psychological debt, and cognitive function, were included. In addition, participants with an AD diagnosis were excluded, resulting in a final sample size of *n* = 298. More information on the quality control process of the neuroimaging data can be found in the DELCODE study protocol [33]. The participant flow chart (Fig S1) and detailed information on the participant selection procedure can be found in the Supplementary Material.

### Measurements

#### Amyloid-beta pathology

The Aβ levels were measured using CSF Aβ42, which was collected in a lumbar punction using the V-PLEX Aβ Peptide Panel 1 (6E10) Kit (K15200E) [33]. To measure Aβ pathology, we used CSF Aβ42 levels. Reduced CSF Aβ42 concentrations reflect early Aβ accumulation and have been shown to precede amyloid PET positivity and predict future Aβ positivity in cognitively unimpaired older adults [34, 35]. In the present study, CSF Aβ42 was inverted by multiplying it with −1 to align its directionality with the other AD-related biomarkers. Higher values of the inverted CSF Aβ42 biomarker indicate greater Aβ pathology.

#### White matter hyperintensities

WMH in the corpus callosum splenium were extracted using isotropic 1mm^3^ high-resolution T2-fluid attenuated inversion recovery (FLAIR) images. Details on acquisition, preprocessing, and extraction process can be found in the Supplementary Material, as well as in previous publications of the DELCODE study protocol [33] and by our group [36]. To account for individual differences in brain size and to ensure accurate comparison across subjects, splenial WMH volumes were adjusted by dividing by the total intracranial volume (TIV). Greater splenial WMH volumes indicate greater brain pathology.

#### Hippocampal volume

Hippocampal volume was assessed via structural MRI data. MRI data was acquired at nine DZNE scanning sites using one Prisma system, one Skyra system, three TIM Trio systems, four Verio systems. Details on the preprocessing and extraction procedures can be found in the Supplementary Material and in the DELCODE study protocol [33]. The volumes of the right and left hippocampus were provided in mm^3^. To calculate the total hippocampal volume, the volumes of the right and left hippocampus were summed and converted from mm^3^ to ml by dividing by 1000. The total hippocampal volume was adjusted for TIV and multiplied by –1 to align its direction with the other AD-related biomarkers. Higher values of the inverted total hippocampal volume indicate greater abnormality and are thereafter referred to as hippocampal neurodegeneration.

#### Cognitive reserve

Cognitive reserve is defined as the adaptability of cognitive processes that allows individuals to cope with brain aging or pathology [2]. Cognitive reserve was operationalized as a construct (latent variable), using lifestyle protective factors or proxies related to education, occupational complexity, and leisure activity participation, as described by Stern and colleagues [2]. In the present study, all three proxies were measured using the Lifetime of Experiences Questionnaire (LEQ), the version adapted for the German population (LEQ-D), as implemented in the DELCODE study [33]. The LEQ measures education, occupational complexity, and long-term engagement in leisure activities at different life stages: young adulthood (13–30 years), mid-life (30–65 years), and late-life (65 + years). Within each life stage, the LEQ can be divided into specific (education and occupational complexity) and non-specific (leisure activities) items. To model cognitive reserve, the unweighted LEQ young adulthood specific score (education), unweighted LEQ mid-life specific score (occupational complexity), and the LEQ mid-life unspecific score (leisure activities) were used. More details can be found in the Supplementary Material. Higher values on this construct (latent variable) are interpreted to reflect higher cognitive reserve.

#### Psychological debt

Psychological debt (previously termed cognitive debt) is defined by the accumulated impact of psychological risk factors including clinical symptoms, personality traits, and adverse life experiences that increase the individual’s vulnerability to cognitive decline and AD [3]. Marchant and Howard [3] highlighted psychological risk factors including depression, anxiety, neuroticism, psychologically-driven sleep disorders, life stress, and post-traumatic stress disorder, that may increase psychological debt. The psychological debt hypothesis posits that these psychological risk factors may work through shared underlying psychological processes. Within this theoretical framework, repetitive negative thinking is proposed as a shared observable transdiagnostic process that drives psychological debt and thereby increases the risk of cognitive decline and AD [3].

In the present SEM study, we aimed to investigate an empirical extension of the psychological debt framework. Specifically, psychological debt was operationalized as a construct (latent variable), represented by four out of the six observable proxies described previously [3]. Modeling psychological debt as a latent variable allowed for the estimation of the shared variance among the proxy indicators while accounting for measurement error. Using this approach, we intended to capture a shared psychological process hypothesized to underlie the psychological risk factors. Psychological risk factors were assessed using validated self-reported questionnaires measuring depressive symptoms (Geriatric Depression Scale), anxiety (Geriatric Anxiety Scale – Short Form), neuroticism (Big Five Inventory), and sleep quality (Pittsburgh Sleep Quality Index). Information on life stress and post-traumatic stress disorder were not available in the DELCODE study [33]. More details on the questionnaires can be found in the Supplementary Material. Higher values on this construct (latent variable) are interpreted to reflect higher psychological debt.

#### Cognitive function

Cognitive function was operationalized as a construct (latent variable) in the SEM, based on the Preclinical Alzheimer’s Cognitive Composite 5 (PACC5) [37] neuropsychological tests, a sensitive cognitive marker for detecting and tracking early cognitive decline associated with AD [37, 38]. The neuropsychological tests included in the PACC5 were adapted from neuropsychological test battery of the DELCODE study [33] and include: Free and Cued Selective Reminding Test—Free + Total Recall (FCSRT-IR), Mini Mental State Examination (MMSE), Wechsler Memory Scale-Revised—Logical Memory Delayed Recall Story A (WMS-R), Symbol-Digit-Modalities Test (SDMT), and the sum of two category fluency tasks. Higher cognitive function on this construct (latent variable) indicates better cognitive function. More details can be found in the Supplementary Material.

#### Risk groups

Information on self-reported sex, collected as part of the demographic data, and APOE ε4 genotype are available in the DELCODE study database. APOE ε4 carrier status was determined through APOE genotyping. Participants carrying at least one ε4 allele (i.e., APOE ε2/4, APOE ε3/4 or APOE ε4/4) were classified as APOE ε4 carriers. Genotyping was performed by identifying the single nucleotide polymorphisms (SNPs) at rs7412 and rs429358 using commercially available TaqMan® SNP Genotyping Assay (ThermoFisher Scientific). More details are provided in the DELCODE study protocol [33].

### Statistical analysis

All statistical analyses were conducted in R (version 4.4.3) with R-Studio (version 2024.09.0) and all statistical scripts are available on Open Science Framework (OSF; https://osf.io/6pfgx/). Descriptive statistics were analyzed using the R package *psych* (version 2.5.3) [39]. To test for group differences in the descriptive statistics, standardized mean difference was calculated using the R package *smd* (version 0.8.0) [40]. The standardized mean difference was calculated as the mean difference divided by the pooled standard deviation. Values of 0.2, 0.5, and 0.8 indicate small, medium, and large differences. The SEM was calculated using the R package *lavaan* (version 0.6–19) [41]. *p*-values < 0.05 were considered statistically significant.

#### Structural equation modeling

Based on previous studies [22, 23, 26], we used SEM to investigate both direct and indirect associations between the constructs (latent variables) of cognitive reserve and psychological debt, observed AD-related biomarkers (CSF Aβ42 [inverted], splenial WMH, hippocampal neurodegeneration), and the construct (latent variable) of cognitive function. In the present structural equation model, the AD-related biomarkers acted as mediators. The following SEM statistical assumptions were assessed for all variables prior to the analysis: multicollinearity and multivariate normality. Standard coefficients (β), *p*-values, and 95% confidence intervals (CI) will be reported later. For more details see Supplementary Material.

Model fit was evaluated using multiple complementary indices reflecting absolute, incremental, and residual-based model fit. The chi-square statistic tests the exact-fit hypothesis, with a non-significant result indicating no statistically detectable discrepancy between the observed and model-implied covariance matrices; however, chi-square is known to be sensitive to sample size and minor model misspecifications. The Root Mean Square Error of Approximation (RMSEA) assesses approximate model misfit per degree of freedom, with values < 0.05 indicating close fit and values between 0.05 and 0.08 indicating reasonable fit. The Standardized Root Mean Square Residual (SRMR) reflects the average standardized residual difference between observed and predicted correlations, with values < 0.08 considered acceptable. Incremental fit was evaluated using the Comparative Fit Index (CFI) and the Tucker-Lewis Index (TLI), which compare the specified model to a null (independence) model; values ≥ 0.90 are commonly interpreted as indicating acceptable fit. These thresholds were treated as general guidelines rather than strict decision rules, and overall model adequacy was evaluated based on the pattern of fit indices in combination.

**Fig. 1:**
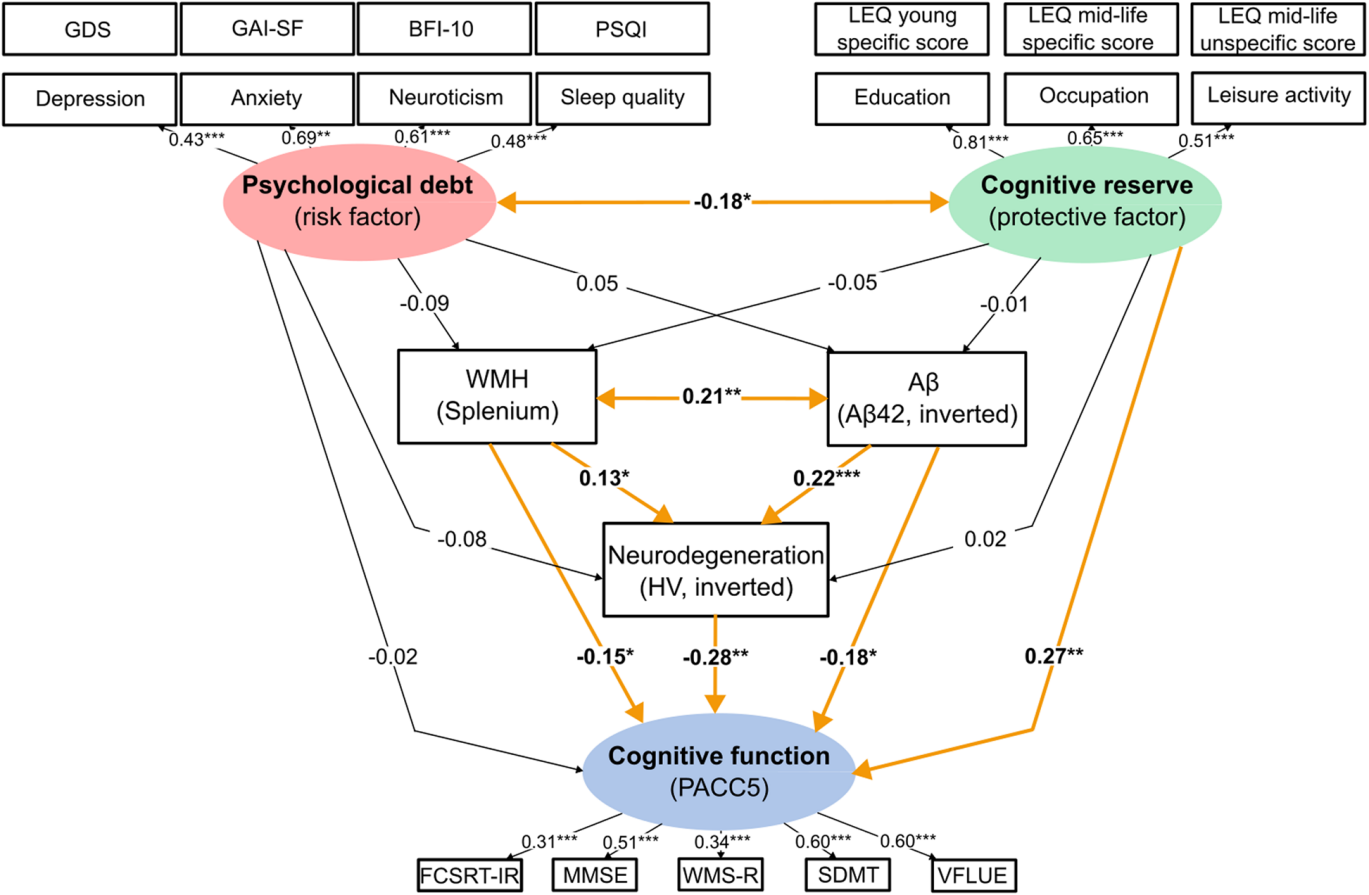
Path diagram of the structural equation model with results of direct associations. The SEM diagram illustrates the associations between the cognitive reserve (shown in green) and psychological debt (shown in red), AD-related biomarkers (CSF Aβ42 [inverted], splenial WMH, hippocampal neurodegeneration), and cognitive function (shown in blue). The structural equation model was adjusted for age. Significant direct associations were observed between cognitive reserve and cognitive function, between AD-related biomarkers and cognitive function, as well as amongst the AD-related biomarkers. All regression analyses were adjusted for age (not shown) as confounding variable. Constructs (latent variables) are represented by ellipses and observed variables are represented by rectangles. Terms show standardized coefficients (β). Significant terms are indicated in bold fonts. Significant paths are indicated with bold lines and in orange. Abbreviations: Aβ = amyloid-beta, AD = Alzheimer’s disease, BFI-10 = Big Five Inventory, CSF = cerebrospinal fluid, FCSRT-IR = Free and Cued Selective Reminding Test—Free + Total Recall, GAI-SF = Geriatric Anxiety Scale – Short Form, GDS = Geriatric Depression Scale, HV = hippocampal volume, LEQ = Lifetime of Experiences Questionnaire, MMSE = Mini Mental State Examination, PACC5 = Preclinical Alzheimer’s Cognitive Composite 5, PSQI = Pittsburg Sleep Quality Index, SDMT = Symbol-Digit-Modalities Test, SEM = structural equation modeling, VFLUE = category fluency test, WMH = white matter hyperintensities, WMS-R = Wechsler Memory Scale-Revised—Logical Memory Delayed Recall Story A. ***p <.001, **p <.01, *p <.05

All variables in the structural equation model were z-standardized. Covariances were estimated between CSF Aβ42 (inverted) and splenial WMH, as well as between cognitive reserve and psychological debt. All regression analyses were adjusted for age as confounding variable. As some variables showed violations of multivariate normality, assumptions were checked using scatterplots. To address non-normality, maximum likelihood with robust standard errors and a mean- and variance-adjusted test statistic using a scale-shifted approach (*mlmv*) was used.The full path model can be found in Fig. 1.

#### Sensitivity analysis

To evaluate the robustness of the associations identified in our structural equation models, we conducted several sensitivity analyses as follows: First, we replaced CSF Aβ42 (inverted) in the main model with the CSF Aβ42/40 ratio (inverted) to assess whether the observed direct and indirect associations with cognitive function would be maintained. Second, we re-calculated the main model in participants with SCD and MCI to assess whether the observed association between cognitive reserve and cognitive function would be maintained in the context of incipient cognitive decline. This approach aligns with the notion that reserve mechanisms become increasingly relevant in the presence of greater pathological burden [42].

#### Multigroup analysis

To investigate differences related to sex and APOE ε4 status in the present structural equation model, multigroup analyses were conducted using (partial) measurement and structural invariance testing. This included establishing configural, metric, scalar, and structural invariance based on the Likelihood Ratio Test. A reduced version of the structural equation model (without testing indirect effects) was specified and fitted using sex (women vs. men) and APOE ε4 status (carriers vs. non-carriers) as the grouping variable, and model fit indices were evaluated. Detailed information on (partial) measurement and structural invariance testing can be found in the Supplementary Material.

## Results

### Sample characteristics

The final sample consisted of a total of *n* = 298 non-demented older participants from the baseline dataset of the DELCODE study (DRKS00007966) [33], with slightly more men (56%) than women (44%). Demographic information of the sample can be found in Table 1. Demographic data stratified by sex and APOE ε4 status groups are provided in the Supplementary Material (see Table S1). To further characterize the demographic composition of the present sample, we provide selected descriptive characteristics of those participants selected and not selected into the present study, as well as of the overall DELCODE study in the Supplementary Material (see Table S2).

**Table 1:** Demographics of the final selected sample of the DELCODE study

	Total sample *M (SD)*	Range^p^ (min. – max.)
Demographics and genetics		
*n*	298	
Age (in years)	69.5 (5.6)	60.0—83.0
Education (in years)	14.8 (2.9)	8.0—20.0
Diagnosis group (*n [%]*): HC, Relatives, SCD, MCI^a^	80 (27%)/ 31 (10%)/ 129 (43%)/ 58 (20%)	-
CSF Aβ42 (*n [%]*): positive/negative^b^	120 (40%)/ 178 (60%)	-
APOE ε4 (*n [%]*): carriers/non-carriers^c^	94 (32%)/ 204 (68%)	-
Sex (n [%]): women/men	131 (44%)/ 167 (56%)	-
Cognitive reserve		
LEQ early life Education^d^	18.5 (5.4)	8.0–32.0
LEQ mid-life Occupational complexity^e^	32.7 (10.9)	0–49.0
LEQ mid-life Leisure activity^f^	18.3 (4.9)	7.0–33.0
Psychological debt		
Depression^g^	1.7 (1.9)	0–10.0
Anxiety^h^	1.0 (1.1)	0–5.0
Neuroticism^i^	2.9 (0.9)	1.0—5.0
Sleep quality^j^	5.3 (3.3)	0–17.0
AD-related biomarkers		
CSF Aβ42^k^	766.6 (315.3)	152.6—1,825.9
CSF Aβ42/40 ratio^l^	0.1 (0.0)	0—0.2
Splenial WMH (in ml)^m^	0.1 (0.1)	0—0.9
Hippocampal volume (in mm^3^)^n^	6,116.2 (790.1)	3,423.9—8,716.6
Cognitive function		
PACC5^o^	−0.3 (0.9)	−3.8—1.9

*Abbreviations*: *Aβ* Amyloid-beta, *AD* Alzheimer’s disease, *APOE ε4* Apolipoprotein ε4, *CSF* Cerebrospinal fluid, *HC* Healthy controls, *LEQ* Lifetime of Experiences Questionnaire, *M* Mean, *MCI* Mild cognitive impairments, *PACC5* Preclinical Alzheimer’s Cognitive Composite 5, *SCD* Subjective cognitive decline, *SD* Standard deviation, *WMH* White matter hyperintensities

^a^Relatives are participants with a family history of AD

^b^Threshold to determine CSF Aβ42 positivity was ≤ 638.7 ( [43])

^c^Participants wit at least one APOE ε4 allele were categorized as APOE ε4 carriers

^d^Measured using the unweighted LEQ young adulthood specific score. Higher scores indicate higher education

^e^Measured using the unweighted LEQ mid-life specific score. Higher scores indicate greater occupational complexity

^f^Measured using the LEQ mid-life unspecific score. Higher scores indicate more engagement in leisure activities

^g^Measured using the Geriatric Depression Scale. Higher scores indicate greater depression symptoms

^h^Measured using the Short Form Geriatric Anxiety Scale. Higher scores indicate greater anxiety symptoms

^i^Measured using the Big Five Inventory—Neuroticism Subscale. Higher scores indicate greater neuroticism

^j^Measured using the Pittsburgh Sleep Quality Index. Higher scores indicate worse sleep quality

^k^Lower scores indicate higher Aβ levels

^l^Lower scores indicate higher Aβ levels

^m^Unadjusted for intracranial volume. Higher scores indicate more splenial WMH

^n^Unadjusted for intracranial volume. Lower scores indicate more hippocampal neurodegeneration

^o^Measured using the observed Preclinical Alzheimer Cognitive Composite Score (PACC). Higher scores indicate better cognitive function

^p^Range of scores in the selected sample

### Structural equation modeling

The structural equation model included the hypothesized associations between cognitive reserve, psychological debt, AD-related biomarkers (CSF Aβ42 [inverted], splenial WMH, hippocampal neurodegeneration), and cognitive function. The model fit was acceptable: chi-square (164.3, *p <* 0.001), RMSEA of 0.05, SRMR of 0.06, CFI of 0.89, and TLI of 0.85.

The structural equation model yielded direct associations shown in Fig. 1 and Table 2 and indirect associations shown in Table 3. Higher cognitive reserve was significantly associated with better cognitive function (β = 0.27, *p* = 0.005; 95% CI: 0.14, 0.41). The AD-related biomarkers were significantly associated with lower cognitive function, independently of one another, for CSF Aβ42 (inverted; β = −0.18, *p* = 0.021; 95% CI: −0.30, −0.06), splenial WMH (β = −0.15, *p* = 0.044; 95% CI: −0.29, −0.00), and hippocampal neurodegeneration (β = −0.28, *p* = 0.007; 95% CI: −0.41, −0.15). The association between higher CSF Aβ42 (inverted) and lower cognitive function was mediated by hippocampal neurodegeneration (indirect effect: β = −0.06, *p* = 0.039; 95% CI: −0.10, −0.02). The AD-related biomarkers were significantly positively correlated with each other (all *p* ≤ 0.029). In addition, we observed a significant negative association between cognitive reserve and psychological debt, such that higher cognitive reserve was correlated with lower psychological debt (β = −0.18, *p* = 0.035; 95% CI: −0.34, −0.02). No significant associations between cognitive reserve and the AD-related biomarkers were observed (all *p* ≥ 0.492). No significant associations were observed between psychological debt and any other variables (all *p* ≥ 0.177).

**Table 2:** Direct associations of the structural equation model

Path	Standardized coefficient (β)	*p*-value
Cognitive reserve → CSF Aβ42 (inverted)	−0.01	.832
Cognitive reserve → splenial WMH	−0.05	.492
Cognitive reserve → hippocampal neurodegeneration	0.02	.705
Cognitive reserve→ cognitive function	0.27	.005**
Cognitive reserve → psychological debt	−0.18	.035*
Psychological debt → CSF Aβ42 (inverted)	0.05	.493
Psychological debt → splenial WMH	−0.09	.177
Psychological debt → hippocampal neurodegeneration	−0.08	.238
Psychological debt → cognitive function	−0.02	.763
CSF Aβ42 (inverted) → hippocampal neurodegeneration	0.22	<.001***
CSF Aβ42 (inverted) → splenial WMH	0.21	.001**
CSF Aβ42 (inverted) → cognitive function	−0.18	.021*
Splenial WMH → hippocampal neurodegeneration	0.13	.029*
Splenial WMH → cognitive function	−0.15	.044*
Hippocampal neurodegeneration → cognitive function	−0.28	.007**

CSF Aβ42 and hippocampal neurodegeneration were inverted before being included in the analysis. Higher scores indicate greater CSF Aβ42 or greater hippocampal neurodegeneration. Significant indirect effects are presented in bold font

*Aβ* Amyloid-beta, *CSF* Cerebrospinal fluid, *WMH* White matter hyperintensities

^*^p <.05, ** *p <*.01, *** *p <*.001

**Table 3:** Indirect associations of the structural equation model

Path	Standardized coefficient (β)	*p*-value
Cognitive reserve → CSF Aβ42 (inverted) → cognitive function	0.002	.834
Cognitive reserve → splenial WMH → cognitive function	0.01	.471
Cognitive reserve → hippocampal neurodegeneration → cognitive function	−0.01	.706
Psychological debt → CSF Aβ42 (inverted) → cognitive function	−0.01	.523
Psychological debt → splenial WMH → cognitive function	0.01	.272
Psychological debt → hippocampal neurodegeneration → cognitive function	0.02	.266
CSF Aβ42 (inverted) → hippocampal neurodegeneration → cognitive function	**−0.06**	.039*
Splenial WMH → hippocampal neurodegeneration → cognitive function	−0.04	.092

CSF Aβ42 and hippocampal neurodegeneration were inverted before being included in the analysis. Higher scores indicate greater CSF Aβ42 or greater hippocampal neurodegeneration. Significant indirect effects are presented in bold font

*Aβ* Amyloid-beta, *CSF* cerebrospinal fluid, *WMH* white matter hyperintensities

^*^p <.05

### Sensitivity analyses

Sensitivity analyses were conducted using the CSF Aβ42/40 ratio (inverted). There was no significant direct association between CSF Aβ42/40 ratio (inverted) and cognitive function (*p* = 0.108). The indirect association between the CSF Aβ42/40 ratio (inverted) and cognitive function via hippocampal neurodegeneration was maintained (β = −0.07, *p* = 0.030; 95% CI: −0.12, −0.02). Next, we re-calculated the main model (with CSF Aβ42 [inverted]) in participants with SCD and MCI. The direct association between cognitive reserve and cognitive function was maintained (β = 0.27, *p* = 0.039; 95% CI: 0.14, 0.41). More detailed results can be found in the Supplementary Material.

### Multigroup analyses

#### Sex (women vs. men)

**Fig. 2:**
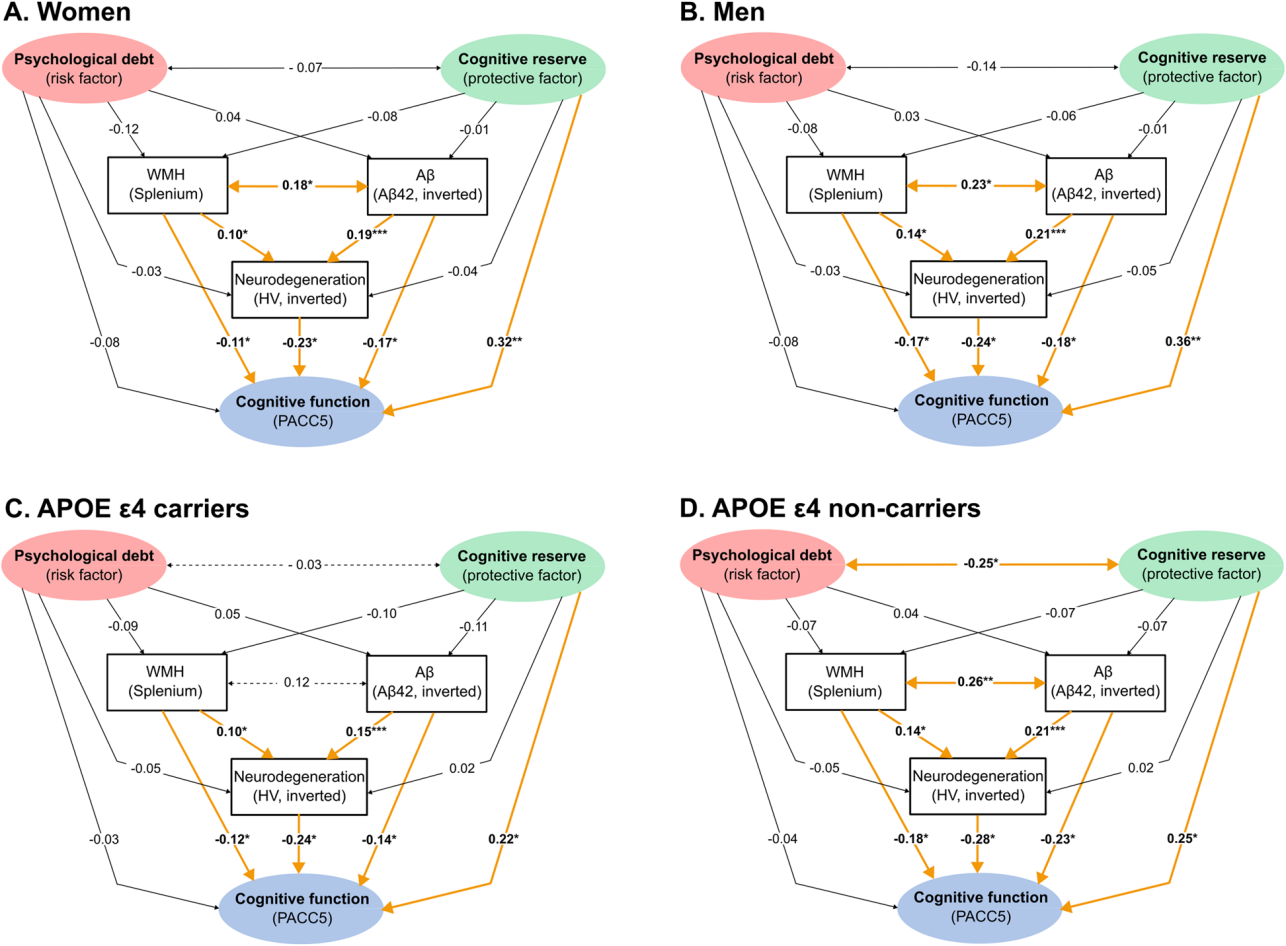
Path diagram of the structural equation model with sex and APOE ε4 status differences (multigroup comparisons). The SEM diagrams illustrate the associations between cognitive reserve (shown in green) and psychological debt (shown in red), AD-related biomarkers (CSF Aβ42 [inverted], splenial WMH, hippocampal neurodegeneration), and cognitive function (shown in blue) in women (**A**) vs. men (**B**) and in APOE ε4 carriers (**C**) vs. non-carriers (**D**). No significant differences between women and men were observed. Significant differences between APOE ε4 carriers vs. non-carriers were observed in the correlations between cognitive reserve and psychological debt as well as between splenial WMH and CSF Aβ42. All regression analyses were adjusted for age (not shown) as confounding variable. Constructs (latent variables) are represented by ellipses and observed variables are represented by rectangles. Terms show standardized coefficients (β). Significant terms are indicated in bold fonts. Significant paths are indicated with bold lines and in orange. Differences between the groups are indicated with scattered lines. Abbreviations: Aβ = amyloid-beta, AD = Alzheimer’s disease, APOE ε4 = apolipoprotein ε4, CSF = cerebrospinal fluid, HV = hippocampal volume, PACC5 = Preclinical Alzheimer’s Cognitive Composite 5, SEM = structural equation modeling. ***p <.001, **p <.01, *p <.05

Multigroup SEM analyses using (partial) measurement and structural invariance testing based on Likelihood Ratio Test were conducted. The model fit of the unconstrained/configural model was acceptable: chi-square (total: 271.6; women: 113.9, men: 112.7, *p <* 0.001; Δchi-square: 32.8), RMSEA of 0.06 (ΔRMSEA: 0.00), SRMR of 0.07 (ΔSRMR: 0.01), CFI of 0.86 (ΔCFI: 0.00), and TLI of 0.81 (ΔTLI: 0.03). Since there was a significant difference between metric and scalar invariance models (*p <* 0.001), we performed partial scalar invariance testing. Therefore, some item intercepts were allowed to differ between groups to maintain a better model fit. The Likelihood Ratio Test (global test) revealed no significant differences in the regression paths between women and men (*p* = 0.599). Direct associations of the partial structural invariance model are shown in Fig. 2. Follow-up Wald tests confirmed no significant sex differences in individual paths (*p* = 0.727).

#### APOE ε4 status (carriers vs. non-carriers)

Multigroup SEM analyses using (partial) measurement and structural invariance testing and Likelihood Ratio Test were conducted. The model fit in the unconstrained/configural model was acceptable: chi-square (total: 267.7; APOE ε4 carriers: 91.5, APOE ε4 non-carriers: 129.6, *p <* 0.001; Δchi-square: 42.6), RMSEA of 0.06 (ΔRMSEA: 0.00), SRMR of 0.07 (ΔSRMR: 0.01), CFI of 0.87 (ΔCFI: 0.01), and TLI of 0.83 (ΔTLI: 0.01). Since there was a significant difference between metric and scalar invariance models (*p <* 0.001), we performed partial scalar invariance testing as above. The Likelihood Ratio Test (global test) revealed significant differences in the regression paths between APOE ε4 carriers vs. non-carriers in the partial structural invariance model (*p* = 0.005). Direct associations of the partial structural invariance model are shown in Fig. 2. In APOE ε4 non-carriers only, the covariances between higher splenial WMH and higher CSF Aβ42 (inverted: β = 0.26, *p* = 0.005; 95% CI: 0.13, 0.39) and between higher cognitive reserve and lower psychological debt (β = −0.25, *p* = 0.020; 95% CI: −0.45, −0.05) were significant.

## Discussion

This cross-sectional study investigated the relationships of cognitive reserve and psychological debt (both modeled as constructs) with AD-related brain pathology (measured by CSF Aβ42, splenial WMH, and hippocampal neurodegeneration), and cognitive function (modeled as a construct based on the PACC5). In non-demented older adults of the DELCODE study [33], we found that higher cognitive reserve was associated with more preserved cognitive function, independent of AD-related biomarkers, while cognitive reserve and psychological debt were not associated with AD-related biomarkers. Each AD-related biomarker was, in turn, independently related to lower cognitive function. These relationships were largely maintained across groups with varying levels of AD risk stratified by sex and APOE ε4 genotype. The APOE ε4 status moderated the associations between cognitive reserve and psychological debt, and between CSF Aβ42 and splenial WMH. Our findings suggest that cognitive reserve may support late-life cognitive resilience, irrespective of AD-related brain pathology and across varying levels of AD risk. The role of psychological debt in these relationships warrants further longitudinal investigation.

### Cognitive reserve and psychological debt, AD-related biomarkers, and cognition

We found that higher cognitive reserve was associated with better cognitive function, independent of concurrent AD-related brain pathology. In our study, cognitive reserve was modeled as a construct (latent variable) encompassing multiple reserve proxies including educational attainment, occupational complexity, and long-term engagement in protective leisure activities. This approach aligns with the conceptualization of cognitive reserve as a theoretical construct that reflects resistance and resilience to brain pathology [2, 44]. Our findings are consistent with a body of previous studies linking lifestyle protective factors, including cognitive and intellectual activities, to more preserved cognitive function in late life [7, 22, 23, 25, 26]. In this SEM study, cognitive function was modeled as a construct based on the PACC5, a sensitive composite known to detect preclinical AD-related cognitive decline [37, 38]. The significant positive association of this latent variable with cognitive reserve was observed despite the presence of AD-related brain pathologies, echoing previous findings [22, 25]. Our observation aligns with findings by Vemuri and colleagues [45], showing that cognitive reserve and AD-related biomarkers independently and additively contribute to cognitive function in older adults. Taken together, our findings and previous research support the view that cognitive reserve and related lifestyle protective factors contribute to cognitive resilience in aging.

Psychological debt was not significantly associated with cognitive function, whereas it showed a significant negative association with cognitive reserve (for discussion see below). The present SEM study is grounded in the psychological debt framework. This framework posits a shared process underlying multiple psychological risk factors [3], with repetitive negative thinking proposed as a key process. Consistent with this, Marchant and colleagues [14] reported that repetitive negative thinking is associated with AD-related brain pathology and faster cognitive decline. In our study, psychological debt was modeled as a construct (latent variable), reflecting the shared vulnerability underlying depressive and anxiety symptoms, neuroticism, and poor sleep quality. The lack of an association of this construct with cognitive function may have several explanations: First, relatively low symptom levels and limited variability in psychiatric measures may have reduced the ability to detect associations. This is convergent with prior findings showing no significant associations between subclinical depressive and anxiety symptoms and baseline cognition [14]. Second, psychological risk factors may be more strongly related to longitudinal cognitive decline than to cross-sectional cognitive function, as documented in other studies [14, 46]. Third, while the latent variable captures shared variance and reduces measurement error, it likely reflects a broader vulnerability profile rather than a specific process. Future research should investigate psychological debt in more vulnerable older populations with mental health symptoms and elucidate the underlying shared mechanisms using combined latent and longitudinal modeling approaches.

We report no significant direct associations between cognitive reserve or psychological debt and the AD-related biomarkers assessed in the present study. While some SEM studies have shown direct associations between lifestyle protective factors as well as psychological risk factors and biomarkers of AD pathology (Aβ and tau burden) and/or white matter lesions [14, 24, 26], this was not found by other studies [22]. The observed null findings may partly reflect limited statistical power and/or the cross-sectional study design may have constrained our ability to detect subtle associations. Alternatively, cognitive reserve and psychological debt may operate through additional neurobiological pathways that were not captured by the present AD-related biomarkers. Existing findings have pointed towards functional connectivity patterns in higher-order brain networks as neural underpinnings of cognitive reserve and psychological debt proxies [47–50], warranting further investigation.

Finally, we show that AD-related biomarkers, reflecting Aβ pathology, splenial WMH, and hippocampal neurodegeneration, were each independently associated with cognitive function, through direct and indirect pathways. The findings of our integrated path model support earlier studies that have linked AD-related brain pathology, as reflected by Aβ biomarkers [22, 25], posterior WMH in the splenium [19], and lower hippocampal volume [22, 24–26], to lower baseline cognitive function and/or faster cognitive decline in older adults. Notably, the present path model demonstrates that each AD-related biomarker uniquely contributes to variability in cognitive function at baseline, corroborating the role of distinct pathological pathways in cognitive aging [51]. Overall, it appears that lifestyle protective factors related to cognitive reserve may contribute to cognitive resilience in late life without necessarily affecting AD-related brain pathology [22], through neurobiological mechanisms and pathways that remain to be determined.

### Risk groups: sex and APOE status

The present findings highlight the relevance of cognitive reserve in late-life cognitive function in older adults with varying levels of AD risk, as defined by sex and APOE ε4 genotype. Specifically, our multigroup analysis showed that the positive relationship between cognitive reserve and cognitive function was maintained across groups with increased risk, namely women and APOE ε4 carriers. This observation supports prior research, showing beneficial effects of lifestyle protective factors regardless of sex or genetic risk [7, 23, 30]. Correspondingly, findings from the *Finnish Geriatric Intervention Study to Prevent Cognitive Impairment and Disability* (FINGER) randomized controlled trial showed that the positive effects of lifestyle interventions on cognitive function did not differ between sexes [7].

We found a moderating role of the APOE ε4 genotype in some risk-related associations, consistent with prior reports of genotype-dependent effects [24, 31, 32, 52]. Specifically, we identified that two associations were moderated by APOE ε4 carrier status, both of which were stronger in non-carriers than in carriers. First, we document a significant negative association between cognitive reserve and psychological debt in the total sample, and this relationship was stronger in non-carriers. This finding mirrors previous studies that have linked higher cognitive reserve to better psychological health or fewer mental health symptoms in adults [53, 54]. In the present study, this relationship was evident in a sample of older adults characterized by subclinical levels of psychological risk factors. Together, these findings highlight the importance of addressing both lifestyle protective factors and psychological risk factors in dementia prevention efforts [55–57]. The fact that the observed negative association was stronger in non-carriers than in carriers might suggest that, among individuals at lower genetic risk, psychological debt may explain a greater proportion of variance. In contrast, in APOE ε4 carriers, increased biological vulnerability may overshadow comparatively subtle influences of psychological risk factors. Alternatively, APOE ε4 carriers may be on a steeper pathophysiological trajectory, potentially attenuating the association between cognitive reserve and psychological debt. Importantly, our findings do not suggest that psychological risk factors are irrelevant in APOE ε4 carriers, but rather that their relative contribution may vary depending on genetic susceptibility.

Secondly, we show that greater splenial WMH were associated with higher Aβ pathology in the total sample, and this relationship was also stronger in non-carriers. The result mirrors previous findings [58] and can be explained by the fact that AD- and vascular-related processes follow partly distinct, though interacting, neuropathological pathways. Posterior WMH have been identified as a region-specific convergence point of AD- and vascular-related pathology, including exposure to hypertension [19–21]. Cerebrovascular alterations, such as impaired cerebral perfusion, blood–brain barrier disruption, or reduced perivascular clearance, may contribute to both WMH and Aβ burden, thereby linking vascular and amyloid pathways. In non-carriers, neuropathology may be more strongly influenced by non-genetic factors, including vascular dysfunction, resulting in a tighter coupling between posterior WMH and Aβ. In contrast, APOE ε4 carriers exhibit a greater amyloid-related biological vulnerability [59], which likely promotes pathogenic pathways through genetically mediated alterations in Aβ aggregation and clearance [60]. These mechanisms may operate partly independent of vascular burden, potentially attenuating the association between posterior WMH and CSF Aβ42 at the phenotypic level [58].

Interestingly, we report no significant moderations by sex. This finding appears to contrast with earlier studies that reported differences between women and men in certain relationships between lifestyle factors, AD-related biomarkers, and cognitive function in older adults [28, 29]. Some variables like metabolic/vascular risk and physical activity that previously showed stronger associations with cognitive function in men compared to women [23], were not included in the present SEM study. Future studies should further investigate sex specificities in these complex relationships in older adults incorporating metabolic/vascular risk factors along with physical activity [23]. Overall, the present findings highlight the importance of stratifying analyses by sex and APOE ε4 carrier status to clarify shared and differential mechanisms and pathways underlying cognitive aging. Longitudinal and mechanistic studies are needed to confirm the present findings and support the development of risk-informed interventions.

### Synopsis and outlook

Taken together, our findings highlight resilience pathways. It appears that promoting a protective lifestyle may support late-life cognitive function, independent of AD-related pathology and across groups with varying levels of AD risk. The observed relationship between cognitive reserve and psychological debt underscores the importance of considering a broader range of psychological factors in research on cognitive aging and dementia [57, 61]. This notion aligns with a recent study, showing that having a psychological profile with lower levels of protective psychological factors (e.g., life-purpose and consciousness) was associated with lower cognitive function in older adults, whereas having a psychological profile with high levels of psychological risk factors was not [62]. In addition, psychological risk factors are frequently assessed in late life in existing cohort studies, although their influences may in fact be stronger in middle life [57]. Given the growing body of evidence suggesting that psychological risk factors contribute to greater vulnerability in aging [1, 8, 63], cohort studies adopting a life-course perspective on potentially modifiable lifestyle and psychological factors are needed. Longitudinal studies should further clarify the temporal dynamics between psychological debt and cognitive decline. This includes disentangling whether psychological debt precedes cognitive decline or whether incipient cognitive decline accelerates psychological debt.

Finally, the investigation of functional brain connectomics may help elucidate the neural underpinnings of cognitive reserve and psychological debt in the aging brain, since protective and risk factors may dynamically shape the organization and functioning of large-scale brain networks [47, 49, 50, 64]. Studies incorporating tau biomarkers [65] may provide further insights into potential relationships between cognitive reserve, psychological debt, and downstream AD-related brain pathology. Future studies should extend the present findings to longitudinal cohorts of more vulnerable older adults, as the role of psychological debt in these complex pathways warrants further investigation.

### Strengths and limitations

Our study has several strengths. 1.) We used SEM rather than single-variable linear regressions to capture the multifactorial nature of AD. By investigating the associations between multiple lifestyle protective and psychological risk factors, AD-related biomarkers, and cognitive function using an integrated path model, we highlight the complex interplay among these factors. 2.) The present sample of the DELCODE study included older participants at along the cognitive continuum, which enhances the generalizability of our findings across different diagnostic stages/groups. 3.) Using a single structural equation model that simultaneously incorporates cognitive reserve, psychological debt, AD-related biomarkers, and cognitive function enabled us to assess the unique contribution of each variable while controlling for the other variables and for age. 4.) Modeling cognitive reserve, psychological debt, and cognitive function as constructs (latent variables) allowed for a more accurate, reliable, and theory-driven assessment of these complex traits. Integrating multiple observed indicators into each construct (latent variable), reduces measurement error and enables more sophisticated statistical modeling.

The following limitations need to be considered. 1.) The present study used cross-sectional data from the DELCODE study, which limits causal interpretations and does not allow conclusions about the directionality of effects. Future studies should examine whether cognitive reserve and psychological debt differ in their effects across diagnostic subgroups, including cognitively unimpaired, SCD, and MCI. In addition, sample characteristics, including specific exclusion criteria (e.g., psychiatric and other clinical conditions), may have limited its representativeness. This may reduce generalizability warranting further investigation in more clinically heterogeneous populations. 2.) Despite the relatively large sample (*N* = 298), model fit was acceptable overall, although some indices fell below guideline thresholds, which is not uncommon in complex structural equation models. Residual-based indices (RMSEA, SRMR) suggested reasonable fit, whereas incremental indices (CFI, TLI) were below recommended cut-offs, indicating marginal fit. This suggests the model does not fully reproduce the observed covariance structure, and results were therefore interpreted with caution. Future studies should replicate our findings with a larger sample size. 3.) Cognitive reserve and psychological debt were operationalized as constructs (latent variables), using retrospective and introspective self-reports using validated questionnaires, which may be prone to reporting bias. While latent variables capture the shared variance among multiple indicators, associations driven by indicator-specific variance may be attenuated, and heterogeneity in effect sizes across indicators may weaken latent-level associations. 4.) Finally, the present study was restricted to a limited set of AD-related biomarkers, namely CSF Aβ42, splenial WMH, and hippocampal volume, which may not fully capture the multifaceted nature of AD pathogenesis. Future studies incorporating an even broader range of AD-related brain pathology are warranted to corroborate and extend the present findings.

## Conclusion

The findings of the present SEM study demonstrate that greater engagement in lifestyle protective factors supporting cognitive reserve is associated with better cognitive function in older adults. This positive relationship was found irrespective of AD-related brain pathology investigated in this study and across different groups with varying levels of AD risk. Our findings highlight the role of lifestyle protective factors in supporting resilience, while the role of psychological debt in these relationships need further investigation. Future research is needed to elucidate underlying neural mechanisms and validate these findings in longitudinal studies.

## Supplementary Information

Supplementary Material 1.

## Data Availability

The data analyzed in this study is subject to the following licenses/restrictions: the pseudonymized data used for this study will be made available by request from any qualified investigator through the DELCODE Steering Board for purposes of replicating procedures and results. Requests to access the minimal dataset should be directed to the German Center for Neurodegenerative Diseases (DZNE), Bonn. For contact information please refer to the DZNE Study Coordination and Project Management (http://www.dzne.de/en/research/studies/clinical-studies/delcode). All statistical scripts used in the present study are available on Open Science Framework (OSF; https://osf.io/6pfgx/).

## Abbreviations

Aβ: Amyloid-beta
AD: Alzheimer’s disease
APOE ε4: Apolipoprotein ε4
CFI: Comparative Fit Index
CI: Confidence interval
CSF: Cerebrospinal fluid
FCSRT-IR: Free and Cued Selective Reminding Test - Free + Total Recall
FLAIR: T2-fluid attenuated inversion recovery images
HV: Hippocampal volume
LEQ: Lifetime of Experiences Questionnaire
LEQ-D: Lifetime of Experiences Questionnaire – German version
M: Mean
MCI: Mild cognitive impairment
mlmv: Maximum likelihood with robust standard errors and a mean- and variance-adjusted test statistic using a scale-shifted approach
MMSE: Mini Mental State Examination
MRI: Magnetic resonance imaging
OSF: Open Science Framework
PACC5: Preclinical Alzheimer’s Cognitive Composite Score
RMSEA: Root mean square error of approximation
SCD: Subjective cognitive decline
SD: Standard deviation
SDMT: Symbol-Digit-Modalities Test
SEM: Structural equation modeling
SNPs: Single nucleotide polymorphisms
SRMR: Standardized root mean square residual
TLI: Tucker-Lewis index
TIV: Total intracranial volume
WMH: White matter hyperintensities
WMS-R: Wechsler Memory Scale-Revised - Logical Memory Delayed Recall Story A
