# Supplementary material for: Pathways to resilience: relationships between cognitive reserve, psychological debt, and Alzheimer’s disease biomarkers
Source: Alzheimers Res Ther. 2026 Apr 24;18:93. doi: 10.1186/s13195-026-02054-z (PMC13107903; doi:10.1186/s13195-026-02054-z)
Supplement: Supplementary file 1 — Supplementary Material 1. [file 13195_2026_2054_MOESM1_ESM.docx]

**Supplementary Material:**

**Pathways to Resilience: Relationships Between Cognitive Reserve, Psychological Debt, and Alzheimer’s Disease Biomarkers**

Hanna Boscheck^#^, Maxie Luft^#^, René Mauer, Selin Senguel, Ylenia D’elia, Peter Dechent, Klaus Fliessbach, Wenzel Glanz, Stefan Hetzer, Daniel Janowitz, Ingo Kilimann, Luca Kleineidam, Marie Kronmüller, Falk Lüsebrink, Lukas Preis, Boris Rauchmann, Ayda Rostamzadeh, Björn Hendrik Schott, Sebastian Sodenkamp, Eike Spruth, Sophia Stöcklein, Renat Yakupov, Gabriel Ziegler, Frederic Brosseron, Katharina Buerger, Julian Hellmann-Regen, Christoph Laske, Robert Perneczky, Oliver Peters, Josef Priller, Alfredo Ramirez, Anja Schneider, Annika Spottke, Stefan Teipel, Jens Wiltfang, Frank Jessen, Emrah Düzel, Sandra Röske, Michael Wagner, Franka Glöckner, Natalie L. Marchant, Olga Klimecki^#^, Miranka Wirth^#^, for the DELCODE study group

# Supplementary methods

## Participant selection procedure

The DELCODE baseline data set comprises *N* = 1079 participants (**Figure S1**). Participants without or unusable Alzheimer’s disease (AD)-related biomarker data (cerebrospinal fluid [CSF] amyloid-beta [Aβ], splenial white matter hyperintensities [WMH], hippocampal volume) were excluded. Additionally, participants without data in the variables of interest to model the two constructs (latent variables) cognitive reserve and psychological debt as well as in cognitive function (Preclinical Alzheimer's Cognitive Composite Score [PACC5]) (1) items were also excluded. Lastly, participants with an AD diagnosis were excluded, resulting in a final sample of *N* = 298 participants.

Insert about here:

=============================================================

**Figure S1****.** Participant flow chart

**
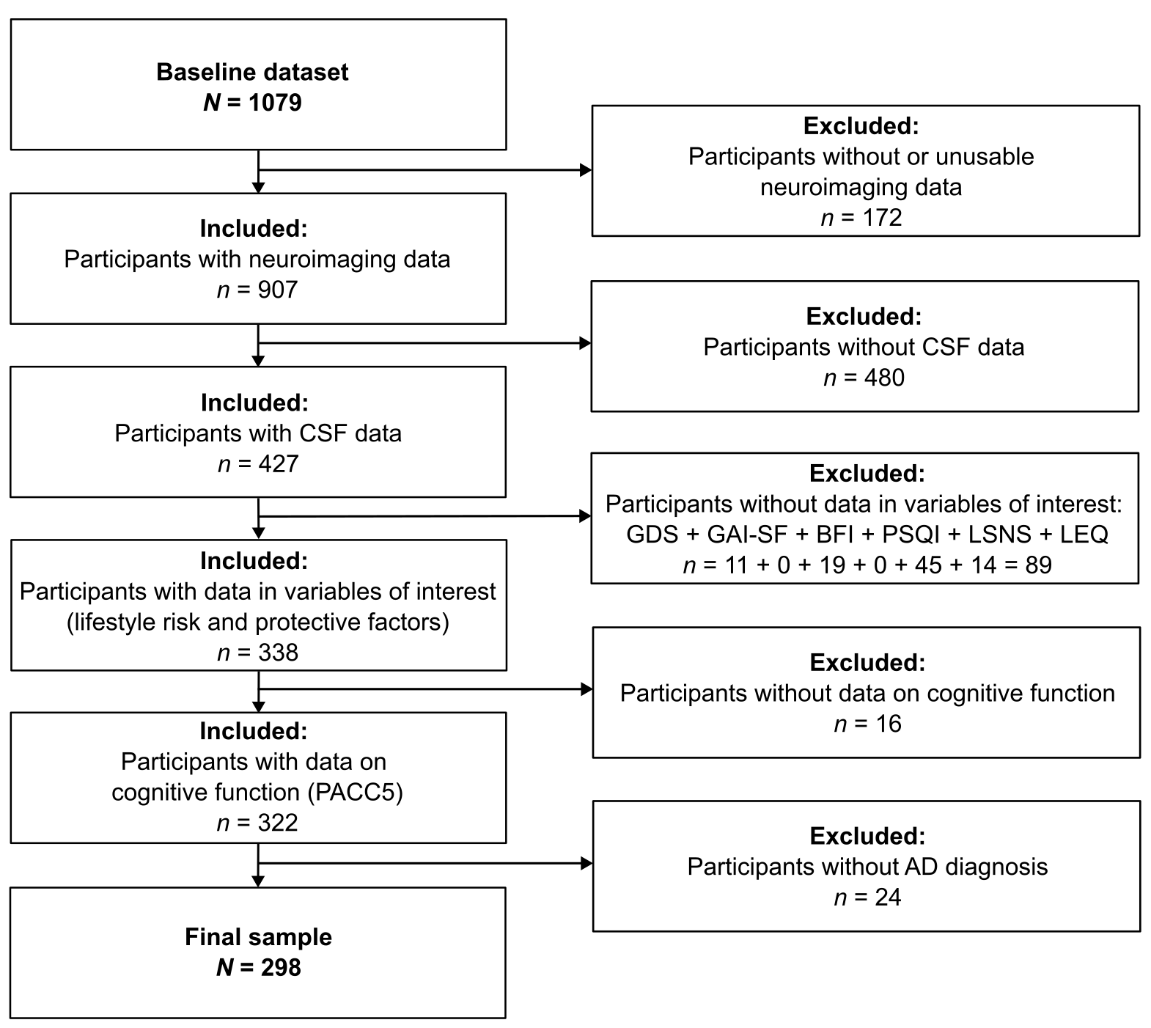
**

*The participant flow chart shows the participant selection process.* *AD = Alzheimer’s disease, BFI Neuroticism = Big Five Inventory - Neuroticism Subscale, CSF = cerebrospinal fluid, GAI-SF = Geriatric Anxiety Scale Short Form, GDS = Geriatric Depression Scale, LEQ = Lifetime of Experiences Questionnaire, LSNS = Lubben Social Networking Scale, PACC5 = Preclinical Alzheimer's Cognitive Composite Scale, PSQI= Pittsburg Sleep Quality Index.*

=============================================================

## Measurements

### White matter hyperintensities

To extract WMH in the splenium of the corpus callosum, T2-FLAIR images with the acquisition parameters repetition time = 5 s, echo time = 394 ms, inversion time = 1.8 s, resolution = 1 × 1 × 1 mm3, matrix = 256 × 256, 192 slices were used. For the WMH segmentation, an automated segmentation algorithm (deep learning algorithm) was used, as described in our previous study (2). WMH were extracted from the region-of-interest (ROI) using the procedure described in our previous study (3). A binary ROI mask delineating the splenium of the corpus callosum was constructed in the Montreal Neurological Institute (MNI) template space and projected onto to the native-space FLAIR images. WMH volumes were calculated within the ROI mask and adjusted for total intracranial volume (TIV).

### Hippocampal volume

In the present study, hippocampal volumes were obtained using an advanced segmentation tool included in FreeSurfer 6.0 (http://surfer.nmr.mgh.harvard.edu/), which enables an automated and more accurate segmentation of hippocampal subfields. This method uses a probabilistic atlas derived from ultra-high-resolution (~0.1 mm isotropic) ex vivo magnetic resonance imaging (MRI) data and incorporates both T1- and high-resolution T2-weighted images when available (4). Specifically, T1-weighted images (3D GRAPPA PAT 2, 1 mm³ isotropic, 256 × 256 px, 192 slices, sagittal, ∼5 min, repetition time = 2500 ms, echo time = 4.33 ms, inversion time = 110 ms, flip angle = 7°) and T2-weighted images optimized for medial temporal lobe volumetry (0.5 × 0.5 × 1.5 mm³, matrix = 384 × 384 px, 64 slices, orthogonal to the hippocampal long axis, ∼12 min, repetition time = 3500 ms, echo time = 353 ms) were used in a multispectral segmentation approach. More details can be found elsewhere (5). Hippocampal volume was adjusted for TIV using the same approach as for WMH.

### Cognitive reserve

In the present study, education was measured using the Lifetime of Experiences Questionnaire (LEQ) (6) using a version adapted to the German population (LEQ-D) (7). In particular, the young adulthood specific score, which assesses the highest level of education by the age of 30 was used. Higher scores indicate higher education. Similarly, occupational complexity was operationalized using the LEQ mid-life specific score, which represents occupational complexity and further educational accomplishments in mid-life, with higher scores indicating higher occupational complexity. Lastly, leisure activities were measured using the LEQ mid-life unspecific score, including several leisure activities, such as social activity, musical activity, artistic activity (e.g., drawing), physical activity, reading, speaking a secondary language, and travel experiences. Higher scores indicate greater engagement in leisure activities.

### Psychological debt

To model the psychological risk construct psychological debt, information on depression, anxiety, neuroticism, and sleep quality were used. To determine depression, the short form of the Geriatric Depression Scale (GDS) (8) was used. The GDS comprises 15 items, with higher scores indicating more severe depressive symptoms. Anxiety was measured using the Short Form Geriatric Anxiety Scale (GAI-SF) (9), which contains 5 items, with higher scores indicating more severe anxiety symptoms. To measure neuroticism, the Neuroticism Subscale of the 10 items Big Five Inventory (BFI-10) (10) was used by calculating the participants’ response to specific items that assess emotional instability and susceptibility to stress, with higher scores indicating greater neuroticism. To measure sleep quality, the Pittsburgh Sleep Quality Index (PSQI) (11) was used that assesses different components of sleep (e.g., quality, latency, duration, efficiency), with higher scores indicating more severe sleep problems (12).

### Cognitive function: Preclinical Alzheimer’s Cognitive Composite Score

Cognitive function was operationalized as a construct (latent variable) using the sub-questionnaires of the PACC5 (1, 13): Free and Cued Selective Reminding Test - Free + Total Recall (FCSRT-IR) (14, 15), Mini Mental State Examination (MMSE) (16), Wechsler Memory Scale-Revised (WMS-R: Logical Memory Delayed Recall Story A) (17), Symbol-Digit-Modalities Test (SDMT) (18), and the sum of two category fluency tasks. The total scores of the individual sub-questionnaires were used to model the construct (latent variable) cognitive function.

## Statistical analysis

All statistical analyses were conducted in R (version 4.4.3) (19) with R-Studio (version 2024.09.0) (20).

### Multigroup analyses

Measurement and structural invariance testing were performed in a stepwise manner. In the first step (configural invariance), no constraints were applied, allowing all parameters to vary freely between the groups. To check the assumptions (applying measurement invariance testing) for testing later the hypothesis (using structural invariance testing), the factors loadings (of the questionnaires) on the constructs (latent variables) were constrained to be equal across the groups to investigate whether the relationships between the observed questionnaires and constructs (latent variables) were equivalent in the second step (metric invariance). In the third step (scalar invariance), in addition to the factor loadings, the intercepts were constrained to test for equivalence in the means of the constructs (latent variables) between the groups**.**

As full scalar invariance could not be established, partial scalar invariance was tested by releasing constraints on specific non-invariant item intercepts. These items were identified using *lavTestScore()* from the R package *lavaan*, which evaluates parameter constraints via score tests. Intercepts that showed significant misfit were freed using the *group.partial* in R function *sem()* in R package *lavaan*, allowing them to vary across groups. This approach enabled meaningful group comparisons despite the absence of full scalar invariance. For hypothesis testing, the regression paths were then constrained to test for group differences in the structural relationships among the constructs (latent variables) in the final step (partial structural invariance)**.** Modell comparisons were performed using the Likelihood Ratio Test (*lavTestLRT()*). Since this global test showed no significant differences between the groups, all paths were additionally tested individually using the Wald test (*lavTestWald()*), which increases sensitivity to subtle effects, as global tests might not detect small group differences in a few individual paths.

# Supplementary results

## Sample characteristics

Additional sample characteristics stratified by sex and apolipoprotein ε4 (APOE ε4) status are provided in **Table S1**. Descriptive data of the overall DELCODE study, the non-selected sub-sample and the selected sub-sample are provided in **Table S2**.

**Table S1.** *Demographics of the final sample stratified by sex and APOE ε4 carrier status.*

|  | **Sex** | | | **APOE status** | | |
| --- | --- | --- | --- | --- | --- | --- |
|  | **Women**  **M (SD)** | **Men**  **M (SD)** | **SMD^p^** | **APOE ε4 carriers**  **M (SD)** | **APOE ε4 non-carriers**  **M (SD)** | **SMD^p^** |
| **Demographics and genetics** | | | | | |  |
| *n* | 131 | 167 | - | 94 | 204 | - |
| Age (in years) | 68.9 (5.2) | 70.0 (5.9) | -0.2 | 69.6 (5.6) | 69.5 (5.6) | 0.0 |
| Sex (n [%]): women/men | - | - | - | 43 (46%)/ 51 (54%) | 88 (43%)/ 116 (57%) | 0.1 |
| Education (in years) | 13.9 (2.8) | 15.6 (2.8) | -0.6 | 15.1 (3.0) | 14.7 (2.9) | 0.2 |
| Diagnosis group (*n [%]*): HC, Relatives, SCD, MCI^a^ | 42 (32%)/ 16 (12%)/ 52 (40%)/ 21 (16%) | 38 (23%)/ 15 (9%)/ 77 (46%)/ 37 (22%) | 0.3 | 18 (19%)/ 10 (11%)/ 43 (46%)/ 23 (25%) | 62 (30%)/ 21 (10%)/ 86 (42%)/ 35 (17%) | 0.3 |
| CSF Aβ42 (*n [%]*): positive/negative^b^ | 48 (37%)/ 83 (63%) | 48 (37%)/ 83 (63%) | 0.1 | 62 (66%)/ 32 (34%) | 58 (28%)/ 146 (72%) | 0.8 |
| APOE ε4 (*n [%]*): carriers/non-carriers^c^ | 43 (33%)/ 88 (67%) | 51 (31%)/ 116 (70%) | 0.0 | - | - | - |
| **Cognitive reserve** |  |  |  |  |  |  |
| LEQ early life Education^d^ | 17.1 (5.0) | 19.7 (5.4) | -0.5 | 18.8 (5.1) | 18.4 (5.5) | 0.1 |
| LEQ mid-life Occupational complexity^e^ | 28.6 (10.3) | 35.9 (10.2) | -0.7 | 32.8 (11.7) | 32.6 (10.5) | 0.0 |
| LEQ mid-life Leisure activity^f^ | 18.1 (4.8) | 18.5 (4.9) | -0.1 | 18.0 (5.1) | 18.4 (4.8) | -0.1 |
| **Psychological debt** |  |  |  |  |  |  |
| Depression^g^ | 1.6 (2.1) | 1.7 (1.8) | -0.0 | 2.0 (2.0) | 1.5 (1.9) | 0.3 |
| Anxiety^h^ | 1.2 (1.2) | 0.8 (1.0) | 0.3 | 1.0 (1.0) | 1.0 (1.1) | -0.0 |
| Neuroticism^i^ | 3.1 (0.9) | 2.7 (0.9) | 0.5 | 3.0 (0.9) | 2.9 (0.9) | 0.2 |
| Sleep quality^j^ | 6.0 (3.5) | 4.8 (3.0) | 0.4 | 5.1 (3.3) | 5.5 (3.3) | -0.1 |
| **AD-related biomarkers** |  |  |  |  |  |  |
| CSF Aβ42^k^ | 792.7 (309.4) | 746.0 (319.3) | 0.1 | 601.6 (250.8) | 842.6 (313.4) | -0.9 |
| CSF Aβ42/40 ratio^l^ | 0.1 (0.0) | 0.1 (0.0) | 0.1 | 0.1 (0.0) | 0.1 (0.0) | -1.1 |
| Splenial WMH (in ml)^m^ | 0.1 (0.1) | 0.1 (0.2) | -0.3 | 0.1 (0.1) | 0.1 (0.1) | -0.0 |
| Hippocampal volume (in mm^3^)^n^ | 5875.0 (736.6) | 6305.4 (781.0) | -0.6 | 5944.8 (906.0) | 6195.1 (719.3) | -0.3 |
| **Cognitive function** |  |  |  |  |  |  |
| PACC5^o^ | -0.1 (0.9) | -0.5 (0.8) | 0.4 | -0.5 (1.1) | -0.2 (0.8) | -0.3 |

*Abbreviations: Aβ = amyloid-beta, AD = Alzheimer’s disease, APOE ε4 = apolipoprotein ε4, CSF = cerebrospinal fluid, HC = healthy controls, LEQ = Lifetime of Experiences Questionnaire, M = mean, MCI = mild cognitive impairments, PACC5 = Preclinical Alzheimer’s Cognitive Composite 5, SCD = subjective cognitive decline, SD = standard deviation, SMD = standardized mean difference, WMH = white matter hyperintensities.*

*^a^Relatives are participants with a family history of AD.*

*^b^Threshold to determine CSF Aβ42 positivity was ≤ 638.7 ((21)).*

*^c^Participants wit at least one APOE ε4 allele were categorized as APOE ε4 carriers.*

*^d^Measured using the unweighted LEQ young adulthood specific score. Higher scores indicate higher education.*

*^e^Measured using the unweighted LEQ mid-life specific score. Higher scores indicate greater occupational complexity.*

*^f^Measured using the LEQ mid-life unspecific score. Higher scores indicate more engagement in leisure activities.*

*^g^Measured using the Geriatric Depression Scale. Higher scores indicate greater depression symptoms.*

*^h^Measured using the Short Form Geriatric Anxiety Scale. Higher scores indicate greater anxiety symptoms.*

*^i^Measured using the Big Five Inventory - Neuroticism Subscale. Higher scores indicate greater neuroticism.*

*^j^Measured using the Pittsburgh Sleep Quality Index. Higher scores indicate worse sleep quality.*

*^k^Lower scores indicate higher Aβ levels.*

*^l^Lower scores indicate higher Aβ levels.*

*^m^Unadjusted for intracranial volume. Higher scores indicate more splenial WMH.*

*^n^Unadjusted for intracranial volume. Lower scores indicate more hippocampal neurodegeneration.*

*^o^Measured using the observed Preclinical Alzheimer Cognitive Composite Score (PACC). Higher scores indicate better cognitive function.
^p^Standardized mean difference (SMD) was calculated as the mean difference divided by the pooled standard deviation. Values of 0.2, 0.5, and 0.8 indicate small, medium, and large differences.*

**Table S2.** *Demographic characteristics of the overall DELCODE study, the non-selected sub-sample, and the selected sub-sample.*

|  | **Total DELCODE study**  **M (SD)** | **Non-selected sub-sample**  **M (SD)** | **Selected sub-sample**  **M (SD)** | **SMD^b^** |
| --- | --- | --- | --- | --- |
| *n* | 1079 | 781 | 298 | - |
| Age (in years) | 70.8 (6.2) | 71.4 (6.3) | 69.5 (5.6) | -0.3 |
| Sex (n [%]): women/men | 550 (51%)/ 529 (49%) | 419 (54%)/ 362 (46%) | 131 (44%)/ 167 (56%) | 0.2 |
| Diagnosis group (*n [%]*): HC, Relatives, SCD, MCI, AD | 236 (22%)/ 82 (8%)/ 445 (41%)/ 190 (18%)/ 126 (12%) | 155 (20%)/ 51 (7%)/ 317 (41%)/ 132 (17%)/ 126 (16%) | 80 (27%)/  31 (10%)/  129 (43%)/ 58 (20%)/ 0 (0%) | 1.1 |
| Education (in years) | 14.4 (3.0) | 14.3 (3.0) | 14.8 (2.9) | 0.2 |
| Depression^a^ | 1.7 (2.0) | 1.7 (2.0) | 1.7 (1.9) | -0.0 |

*Abbreviations: AD = Alzheimer’s disease, HC = healthy controls, M = mean, MCI = mild cognitive impairment, SCD = subjective cognitive decline, SMD = standardized mean difference, SD = standard deviation.*

*^a^Measured using the Geriatric Depression Scale. Higher scores indicate greater depression symptoms.*

*^b^Standardized mean difference (SMD) was calculated as the mean difference divided by the pooled standard deviation. Values of 0.2, 0.5, and 0.8 indicate small, medium, and large differences.*

## Sensitivity analyses

**CSF Aβ42/40 ratio:** First, the CSF Aβ42/40 ratio (inverted) was included in the structural equation model in place of CSF Aβ42 (inverted). The model fit was acceptable: chi-square (173.9, *p* < .001), Root Mean Square Error Approximation (RMSEA) of 0.06, Standardized Root Mean Square Residual (SRMR) of 0.06, Comparative Fit Index (CFI) of 0.88, and Tucker-Lewis Index (TLI) of 0.83. Direct associations are shown in **Figure S2**. The AD-related biomarkers remained positively significantly associated with each other (all *p* ≤ .021). The direct association between CSF Aβ42/40 ratio (inverted) and cognitive function was not significant (*p* = .108). The indirect association between higher CSF Aβ42/40 ratio (inverted) and lower cognitive function mediated by hippocampal neurodegeneration was maintained (β = -0.07, *p* = .030; 95% CI: -0.12, -0.02).

**Figure S2.** *Path diagram of sensitivity analyses with CSF Aβ42/40 ratio as AD-related biomarker with results of direct associations*


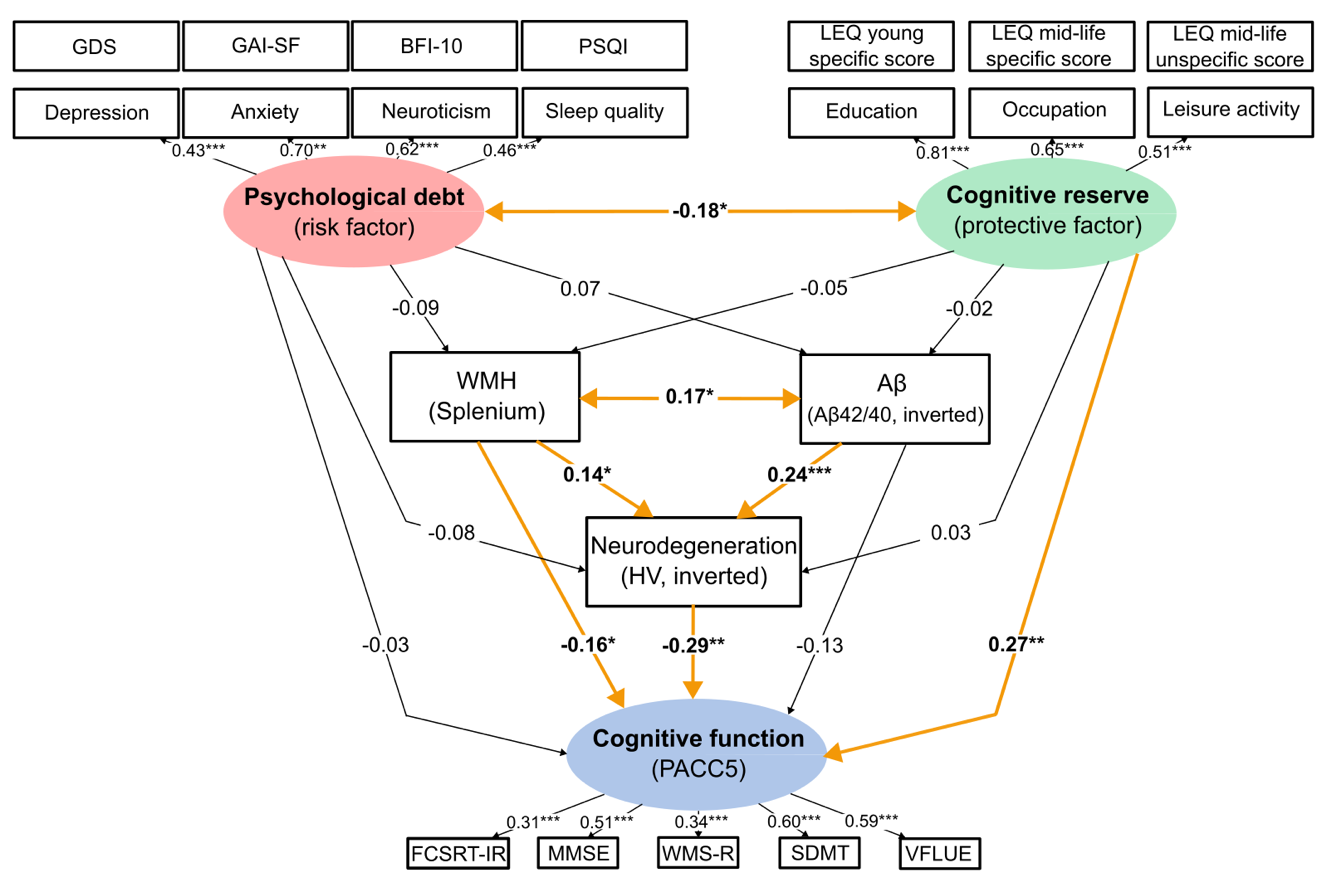


*Associations between the protective and risk constructs cognitive reserve (shown in green) and psychological debt (shown in red), AD-related biomarkers (CSF Aβ42/40 [inverted], splenial WMH, hippocampal neurodegeneration), and the construct (latent variable) cognitive function (PACC5; shown in blue). Constructs (latent variables) are represented by ellipses, while manifest variables are represented by rectangles. Significant direct associations between AD-related biomarkers (except CSF Aβ42/40 [inverted]) and cognitive function, cognitive reserve and cognitive function, as well as between AD-related biomarkers were observed. All regression analyses were adjusted for age (not shown) as confounding variable. Terms show standardized coefficients (ß). Significant terms are indicated in bold fonts. Significant paths are indicated with bold lines and in orange. Aβ = amyloid-beta, AD = Alzheimer’s disease, BFI-10 = Big Five Inventory, CCs = corpus callosum splenium, CSF = cerebrospinal fluid, FCSRT-IR = Free and Cued Selective Reminding Test - Free + Total Recall, GAI-SF = Geriatric Anxiety Scale – Short Form, GDS = Geriatric Depression Scale, HV = hippocampal volume, LEQ = Lifetime of Experiences Questionnaire, MMSE = Mini Mental State Examination, PACC5 = Preclinical Alzheimer’s Cognitive Composite Score, PSQI = Pittsburg Sleep Quality Index, SDMT = Symbol-Digit-Modalities Test, VFLUE = category fluency test, WMH = white matter hyperintensities, WMS-R = Wechsler Memory Scale-Revised - Logical Memory Delayed Recall Story A. ***p < .001, **p < .01, *p < .05.*

## Participants with SCD and MCI: Second, the main structural equation model was calculated in participants with subjective cognitive decline (SCD) and mild cognitive impairment (MCI). The model fit was acceptable: chi-square (150.3, *p* < .001), RMSEA of 0.06, SRMR of 0.07, CFI of 0.86, and TLI of 0.81. The association between cognitive reserve and cognitive function was maintained (β = 0.22, *p* = .039; 95% CI: 0.06, 0.39). The covariance between psychological debt and cognitive reserve was maintained (β = -0.29, *p* = .029; 95% CI: -0.50, -0.08). The associations between CSF Aβ42 (inverted) and splenial WMH (β = 0.23, *p* = .001; 95% CI: 0.13, 0.34) as well as between CSF Aβ42 (inverted) and hippocampal neurodegeneration (β = 0.21, *p* = .002; 95% CI: 0.08, 0.35) were maintained.

# References

1. Papp KV, Rentz DM, Orlovsky I, Sperling RA, Mormino EC. Optimizing the preclinical Alzheimer's cognitive composite with semantic processing: The PACC5. Alzheimers Dement (N Y). 2017;3(4):668-77.

2. Gaubert M, Dell'Orco A, Lange C, Garnier-Crussard A, Zimmermann I, Dyrba M, et al. Performance evaluation of automated white matter hyperintensity segmentation algorithms in a multicenter cohort on cognitive impairment and dementia. Front Psychiatry. 2023;12(13).

3. Gaubert M, Lange C, Garnier-Crussard A, Köbe T, Bougacha S, Gonneaud J, et al. Topographic patterns of white matter hyperintensities are associated with multimodal neuroimaging biomarkers of Alzheimer's disease. Alzheimers Res Ther. 2021;13(1):29.

4. Iglesias JE, Augustinack JC, Nguyen K, Player CM, Player A, Wright M, et al. A computational atlas of the hippocampal formation using ex vivo, ultra-high resolution MRI: Application to adaptive segmentation of in vivo MRI. Neuroimage. 2015;15(115):117-37.

5. Düzel E, Berron D, Schütze H, Cardenas-Blanco A, Metzger C, Betts M, et al. CSF total tau levels are associated with hippocampal novelty irrespective of hippocampal volume. Alzheimers Dement (Amst). 2018;2(10):782-90.

6. Valenzuela MJ, Sachdev P. Assessment of complex mental activity across the lifespan: development of the Lifetime of Experiences Questionnaire (LEQ). Psychological medicine. 2007;37(7):1015-25.

7. Roeske S, Wolfsgruber S, Kleineidam L, Zulka L, Buerger K, Ewers M, et al. P3-591: A German version of the lifetime of experiences questionnaire (LEQ) to measure cognitive reserve: Validation results from the DELCODE study. Alzheimer's & Dementia. 2018;14(7S_Part_25):P1352-P3.

8. Yesavage JA, Brink TL, Rose TL, Lum O, Huang V, Adey M, et al. Development and validation of a geriatric depression screening scale: A preliminary report. Journal of Psychiatric Research. Journal of Psychiatric Research. 1982;17(1):37-49.

9. Byrne GJ, Pachana NA. Development and validation of a short form of the Geriatric Anxiety Inventory – the GAI-SF International Psychogeriatrics. 2011;23:1:125–31.

10. Rammstedt B, John OP. Measuring personality in one minute or less: A 10-item short version of the Big Five Inventory in English and German. Journal of Research in Personality. 2007;41:203-12.

11. Buysse DJ, Reynolds CF, 3rd, Monk TH, Berman SR, Kupfer DJ. The Pittsburgh Sleep Quality Index: a new instrument for psychiatric practice and research. Psychiatry Res. 1989;28(2):193-213.

12. Carpenter JS, Andrykowski MA. Psychometric evaluation of the pittsburgh sleep quality index. Journal of Psychosomatic Research. 1998;45(1):5-13.

13. Wolfsgruber S, Kleineidam L, Weyrauch AS, Barkhoff M, Röske S, Peters O, et al. Relevance of Subjective Cognitive Decline in Older Adults with a First-Degree Family History of Alzheimer's Disease. cline in Older Adults with a First-Degree Family History of Alzheimer's Disease 2022;87(2):545-55.

14. Buschke H. Cued recall in amnesia. J Clin Neuropsychol. 1984;6(4):433-40.

15. Grober E, Buschke H. Genuine memory deficits in dementia. Dev Neuropsychol. 1987;3:13-6.

16. Folstein MF, Folstein SE, McHugh PR. "Mini-mental state". A practical method for grading the cognitive state of patients for the clinician. J Psychiatr Res. 1975;12(3):189-98.

17. Wechsler D. WMS-R: Wechsler Memory Scale--Revised : Manual: Psychological Corporation; 1987.

18. Smith A. Symbol digit modalities test: Manual. Los Angeles, CA: Western Psychological Services; 1982.

19. R Core Team. R: A language and environment for statistical computing. Vienna, Austria: R Foundation for Statistical Computing; 2024.

20. Posit team. RStudio: Integrated development environment for R. Boston, MA: Posit Software, PBC; 2024.

21. Jessen F, Wolfsgruber S, Kleineindam L, Spottke A, Altenstein S, Bartels C, et al. Subjective cognitive decline and stage 2 of Alzheimer disease in patients from memory centers. Alzheimer's & dementia : the journal of the Alzheimer's Association. 2022;19(2):487-97.
